# Porous microspheres support mesenchymal progenitor cell ingrowth and stimulate angiogenesis

**DOI:** 10.1063/1.5008556

**Published:** 2018-04-26

**Authors:** Thomas E. Paterson, Giulia Gigliobianco, Colin Sherborne, Nicola H. Green, James M. Dugan, Sheila MacNeil, Gwendolen C. Reilly, Frederik Claeyssens

**Affiliations:** 1 Department of Materials Science and Engineering, Kroto Research Institute, Broad Lane, University of Sheffield, Sheffield S3 7HQ, United Kingdom; 2 Department of Materials Science and Engineering, Insigneo Institute for in silico Medicine, The University of Sheffield, Sheffield S1 3JD, United Kingdom

## Abstract

Porous microspheres have the potential for use as injectable bone fillers to obviate the need for open surgery. Successful bone fillers must be able to support vascularisation since tissue engineering scaffolds often cease functioning soon after implantation due to a failure to vascularise rapidly. Here, we test the angiogenic potential of a tissue engineered bone filler based on a photocurable acrylate-based high internal phase emulsion (HIPE). Highly porous microspheres were fabricated via two processes, which were compared. One was taken forward and investigated for its ability to support human mesenchymal progenitor cells and angiogenesis in a chorioallantoic membrane (CAM) assay. Porous microspheres with either a narrow or broad size distribution were prepared via a T-junction microfluidic device or by a controlled stirred-tank reactor of the HIPE water in oil in water (w/o/w), respectively. Culture of human embryonic stem cell-derived mesenchymal progenitor (hES-MP) cells showed proliferation over 11 days and formation of cell-microsphere aggregates. *In-vitro*, hES-MP cells were found to migrate into microspheres through their surface pores over time. The presence of osteoblasts, differentiated from the hES-MP cells, was evidenced through the presence of collagen and calcium after 30 days. Microspheres pre-cultured with cells were implanted into CAM for 7 days and compared with control microspheres without pre-cultured cells. The hES-MP seeded microspheres supported greater angiogenesis, as measured by the number of blood vessels and bifurcations, while the empty scaffolds attracted host chick cell ingrowth. This investigation shows that controlled fabrication of porous microspheres has the potential to create an angiogenic, bone filling material for use as a cell delivery vehicle.

## INTRODUCTION

Bone grafts require rapid vascularisation upon implantation to be successful.^1^ Vascularisation of tissue engineered scaffolds is now recognised as a major stumbling block in producing full thickness tissues.^2^ To tissue engineer full thickness tissues or complete organs, rapid vascularization of the scaffold is essential^3^ since the diffusion of oxygen within the tissues becomes the limiting factor (the diffusion limit is normally quoted as 100–200 *μ*m).^4^ Cells that are further away from a blood vessel than the diffusion limit are unable to survive because of hypoxia and undergo necrosis, releasing chemicals and enzymes into the local environment which impedes tissue regeneration.^5^

An important target for tissue engineering large defects is bone, where critical size defects can be treated with synthetic bone fillers, normally putties or pastes that are potentially injectable. These bone fillers will need to rely on rapid *in-situ* angiogenesis, i.e. the stimulation of new blood vessels from existing vasculature^6^ to enable nutrient supply to cells within the implanted filler.^1^ Current tissue engineered solutions for bone defects usually avoid cell-based therapies, depending instead on cells migrating from the periphery of the implantation site.^7,8^ This causes a slow tissue ingrowth starting from the periphery.^7^ To support rapid cell ingrowth and allow vascularisation, an injectable bone filler should ideally be highly porous,^9,10^ and in this study, we investigate highly porous microspheres to achieve both. These porous microspheres can be used for many applications in tissue engineering such as microcarriers for cell expansion,^11^ cell implantation,^12^ delivery of bioactive agents,^13^ and building blocks for (self-assembled) scaffolds.^14,15^ The advantage of using microspheres is that they can be delivered as an injectable substrate, bypassing the requirement for open surgery. As a three-dimensional (3D) cell support matrix for cells, porous microspheres have many advantages over their non-porous counterparts; they can provide enhanced nutrient diffusion, a 3D culture environment, and a greatly increased surface area.^16,17^ There are many techniques to manufacture porous microsphere systems including supercritical CO_2_,^18^ thermally induced phase separation,^19^ freeze thaw cycles,^20^ particle leaching,^21^ and polymerised high internal phase emulsion (polyHIPE) formulations.^22^

PolyHIPE fabrication methods are of particular interest because of the extremely high interconnected porosity achievable with this system. PolyHIPEs (polymers with an open porosity greater than 74% of the total internal volume)^23,24^ can be fashioned into porous microspheres via a double emulsion.^25^ The HIPE emulsion is produced by the dropwise addition of the internal phase to a continuous phase. If the continuous phase is composed of suitable monomers and cross-linkers, a highly porous foam (polyHIPE) can be produced upon curing.^26^ This technique is referred to as the controlled stirred-tank reactor (CSTR) method. The interconnected nature of a polyHIPE is formed by the contraction of the thin monomer film surrounding the droplet phase during curing.^27^ Controlling the processing conditions allows precise control over the degree of porosity within the material along with control over the interconnectivity and to some extent pore size.^28^ We have recently demonstrated that the mechanical properties of this copolymer system can be finely tuned by changing the monomer ratios.^29^ PolyHIPEs are increasingly being used in tissue engineering applications and as cell culture substrates due to their porosity and interconnectivity.^23,30^ However, little is currently known about polyHIPE microspheres' ability to support osteoprogenitor cells or angiogenesis. The aim of this study was to identify an easily controllable manufacturing method for highly porous microsphere scaffolds capable of supporting mesenchymal stem cell (MSC)-like cells and to measure their vascularisation potential using a chorioallantoic membrane (CAM) assay.

## RESULTS

### Control of internal porosity of polyHIPE

**FIG. 1.**
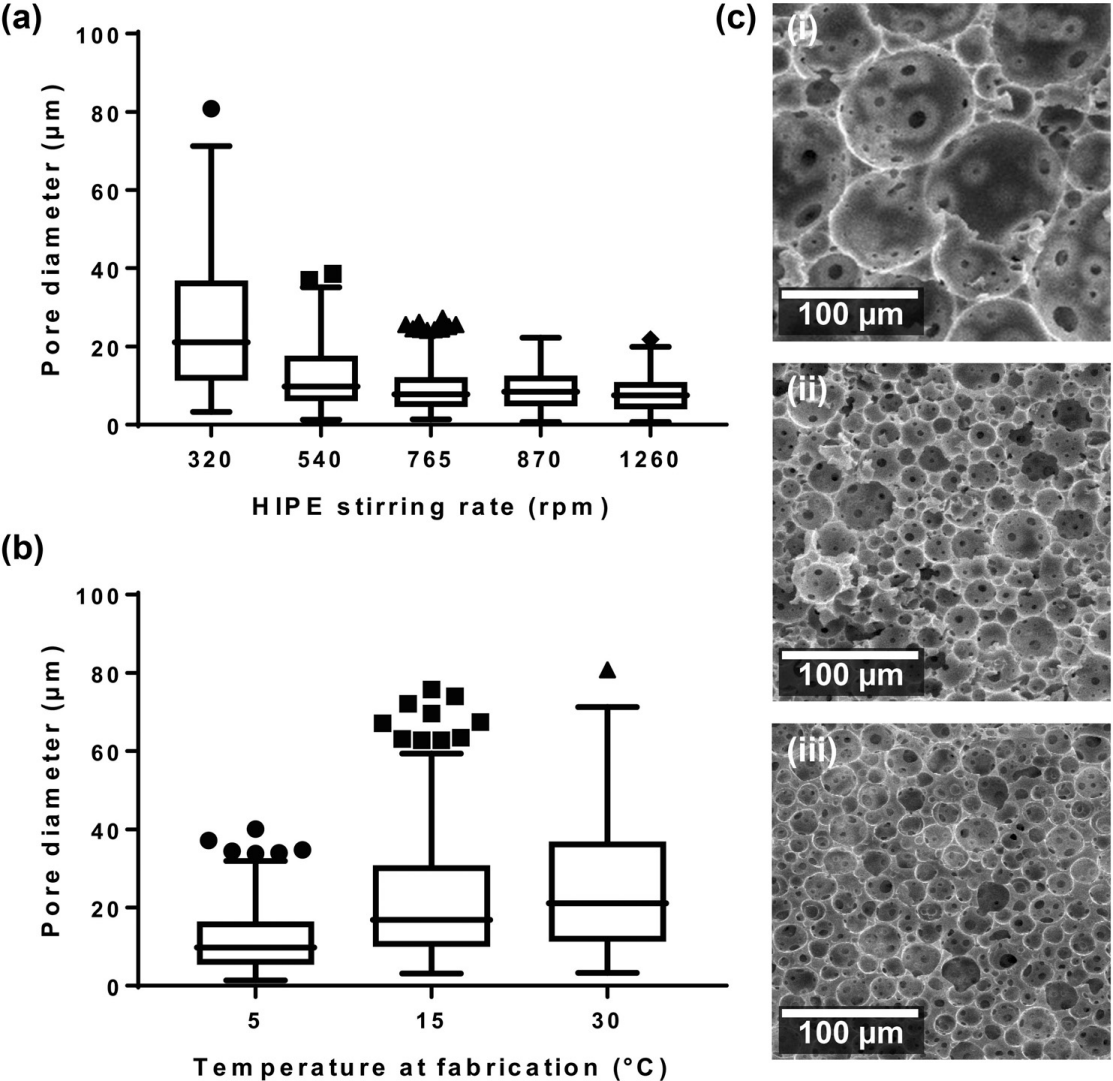
(a) Box plot of the effect of changing the stir rate to produce the o/w emulsion on the pore diameter. With the increasing stirring rate, the median pore diameter within the polyHIPE decreases initially, along with the distribution of pore sizes. After 765 rpm, there is little effect on the pore size with the increasing stir rate. The box plot presents the interquartile range with the centre line as the median, and whiskers represent the ± 1.5 IQR of the microsphere/pore diameter. (b) The relationship between the temperature of the material during HIPE mixing and the resulting pore sizes measured in the polyHIPE is shown on a Tukey boxplot. The temperature of the water added to the monomer is controlled at 4, 14, and 30 °C. (c) SEM micrographs of sectioned polyHIPE blocks to show internal porosity: (c-i) 320 rpm, (c-ii) 765 rpm, and (c-iii) 1260 rpm.

**The internal porosity of the polyHIPE can be controlled via the HIPE *pre-*processing conditions.** The stirring rate and temperature used during the formation of the primary emulsion, or water in oil (w/o) emulsion, had a direct effect on the pore size distribution of the resulting polyHIPE. The pore size and size distribution of the polyHIPE material were measured after the emulsion was formed using different stirring rates and set temperatures (Fig. 1). The biggest difference in pore sizes was between 320 and 540 rpm settings (median pore sizes of 21 *μ*m and 10 *μ*m, respectively), with both the median and the maximum pore size being smaller in the 540 rpm group. An observable decrease in the pore diameter was evident between 540 and 765 rpm (median pore sizes of 10 *μ*m and 8 *μ*m, respectively) although the change is less than that between 320 and 540 rpm. The final 3 speeds of 765, 870, and 1260 rpm yielded similar resulting size distributions. For the material formed at 30 °C with the slowest stir speed of 320 rpm, the largest pore observed was 80 *μ*m in diameter with the pore interconnections measured at a maximum of 6.4 *μ*m diameter with a median of 2 *μ*m. In this study, we choose the manufacturing method to produce the largest pore size, and using the data from Fig. 1, we prepared the emulsion with a mixing speed of 320 rpm at a temperature of 30 °C.

### Porous particle fabrication

**FIG. 2.**
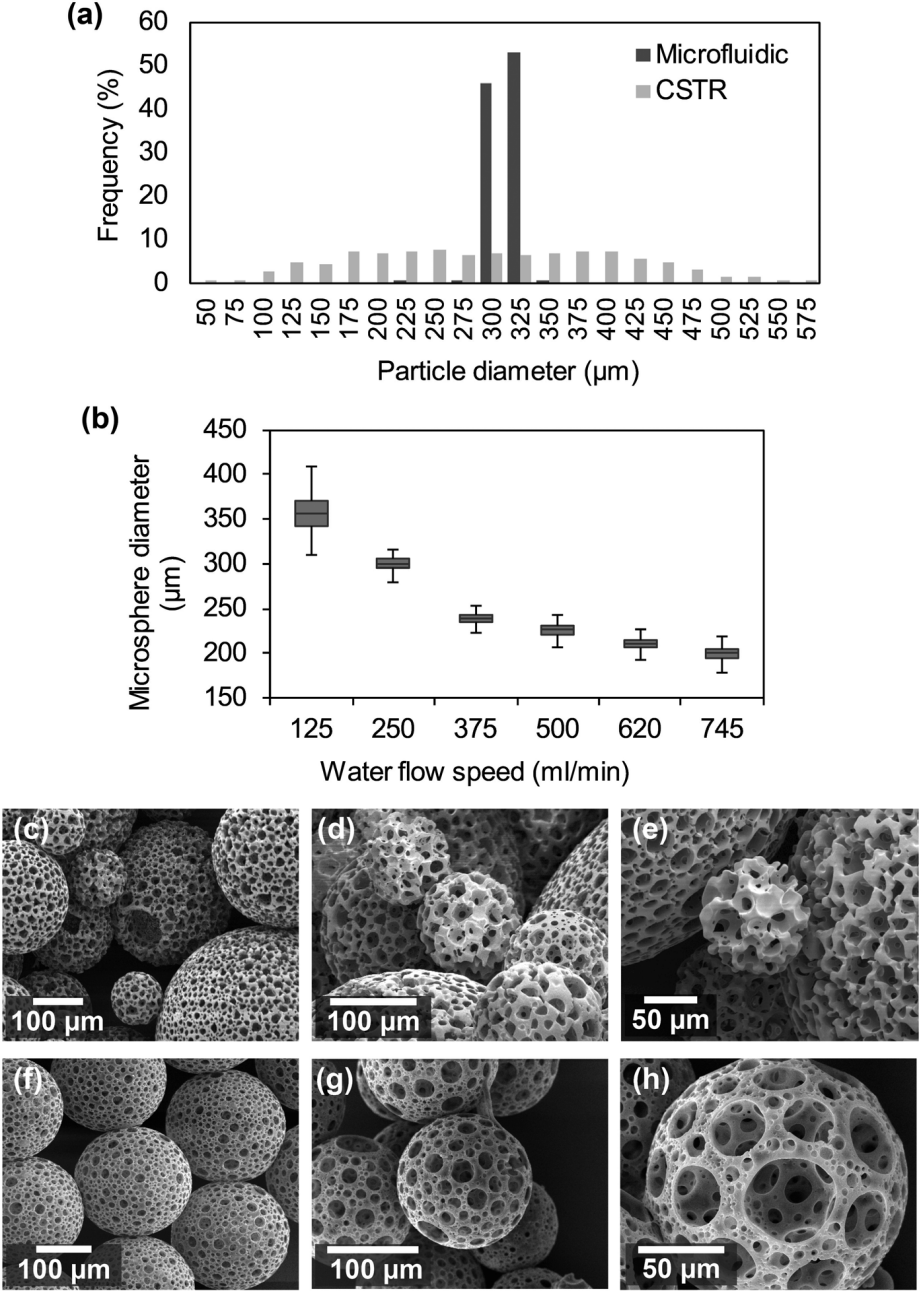
Comparison of the distribution of microsphere sizes formed from two techniques with a similar mean microsphere diameter, EHA/IBOA blend polyHIPE material with 80% porosity and internally interconnected porosity between larger pores. (a) Graph showing the microspheres from the microfluidic technique (narrow size distribution) with microsphere sizes from the CSTR (broad size distribution). (b) Tukey boxplot showing the distribution of microspheres formed via altering the manufacturing parameter of the water flowrate. Microspheres produced from a median diameter of 200 *μ*m using the fastest flow rate of 745 ml/min to 355 *μ*m with the slowest flow rate of 125 ml/min. (c)–(e) PolyHIPE microspheres formed from the CSTR technique. SEM micrographs showing the range of sizes present in a microsphere population produced from this technique. (f)–(h) Microspheres produced by the microfluidic technique. SEM micrographs of microspheres produced by the microfluidic technique, and microspheres can be produced in different sizes with a narrow size distribution.

Porous particles were fabricated via two techniques, either via CSTR or via a microfluidic fabrication route. Microspheres produced by the CSTR technique produce a range of sizes, whereas the microfluidic technique produced microspheres with a narrow size distribution (Fig. 2). Microspheres were formed from a blend of two monomers, 2-ethylhexyl acrylate (EHA) and isobornyl acrylate (IBOA), and photopolymerization was used to polymerise the monomers into microspheres. The bar plot summarises the size distributions from both manufacturing techniques using microsphere populations of similar mean values, CSTR with 286 *μ*m, and the microfluidic with 300 *μ*m [Fig. 2(a)]. The graph shows the wide distribution of microsphere sizes formed by CSTR (26–583 *μ*m) to the narrower distribution formed by T-microfluidics (278–323 *μ*m). Increasing the flow rate of the water allowed control over the size of the resulting microsphere population when using the microfluidic manufacturing technique. Microspheres produced from a median diameter of 200 *μ*m used the fastest flow rate of 745 ml/min to a median 355 *μ*m with the slowest flow rate of 125 ml/min. At slower flow rates, the spread of microsphere sizes began to increase. SEM microscopy was carried out to analyse the microsphere diameter and surface topology on the manufactured microspheres. Figures 2(c)–2(e) present typical SEM images of microspheres produced by CSTR and [Figs. 2(f)–2(h)] highlight microspheres produced by T-junction microfluidic. A difference in surface topography is seen between the two techniques with the microspheres produced by the microfluidic method having a smoother surface than those produced by CSTR which have a rougher surface. For both manufacturing methods, the resulting microsphere surfaces are dominated by largest open pores when considering the volume of pores compared to the number of pores. From this experiment, the parameters were chosen to manufacture microspheres with a median diameter of 200 *μ*m for the ingrowth experiment later in this study.

**FIG. 3.**
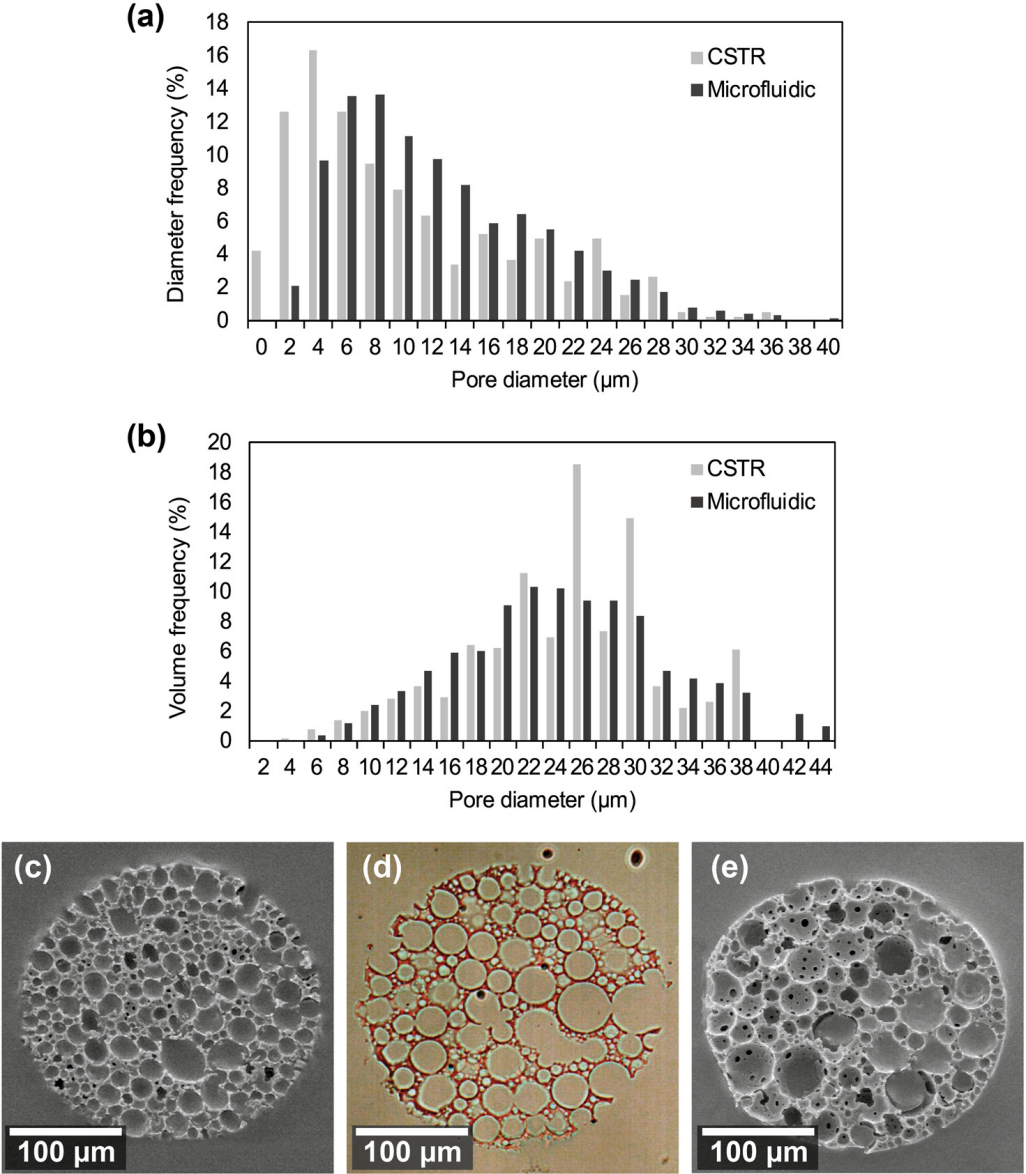
Comparison of the effect of the production technique on the internal porosity of polyHIPE microspheres from the same HIPE monomer batch. (a) Histogram of the distribution of pore diameters inside microspheres formed from microfluidics and CSTR. Microspheres formed from CSTR appeared to have a higher proportion of smaller pores than microspheres from the microfluidic technique. (b) Histogram of pore volumes displaying the volume multiplied by frequency to show the distribution of total volumes at each size pore. (c) SEM micrograph of a sectioned microsphere formed via the CSTR method. (d) Optical image of a 10 *μ*m thick slice of a polyHIPE microsphere formed by CSTR and sectioned using a microtome. Uniform porosity can be observed throughout the microsphere along with a degree of interconnectivity. (e) SEM micrograph of a sectioned microsphere formed via the T-junction microfluidic device.

Both techniques produce microspheres containing internal pores of similar diameter and diameter distribution (Fig. 3). Microspheres produced by both techniques were sectioned and analysed to determine if the manufacturing method influenced the resulting internal pore sizes. The two distributions obtained from each manufacturing method share a similar range of pore sizes with a slight shift to lower pore sizes in those produced via CSTR [Fig. 3(a)]. This difference is seen most strongly at the 4 to 6 *μ*m pore sizes, with these being more abundant in microspheres prepared by CSTR. Therefore, the microfluidic technique was used for microspheres fabricated for further analysis and cell culture. The total volume of the pores against the pore size is shown in Fig. 3(b). Despite a higher incidence of small pores, the overall volume represented remains equal across all pore sizes. SEM and optical images of thin sections of microspheres prepared using a microtome [Figs. 3(c)–3(e)] show that the porosity is consistent throughout the whole structure, and there are regions of interconnectivity between pores.

### Metabolic activity of human embryonic stem cell-derived mesenchymal progenitor (hES-MP) cells cultured on the polyHIPE microspheres

**FIG. 4.**
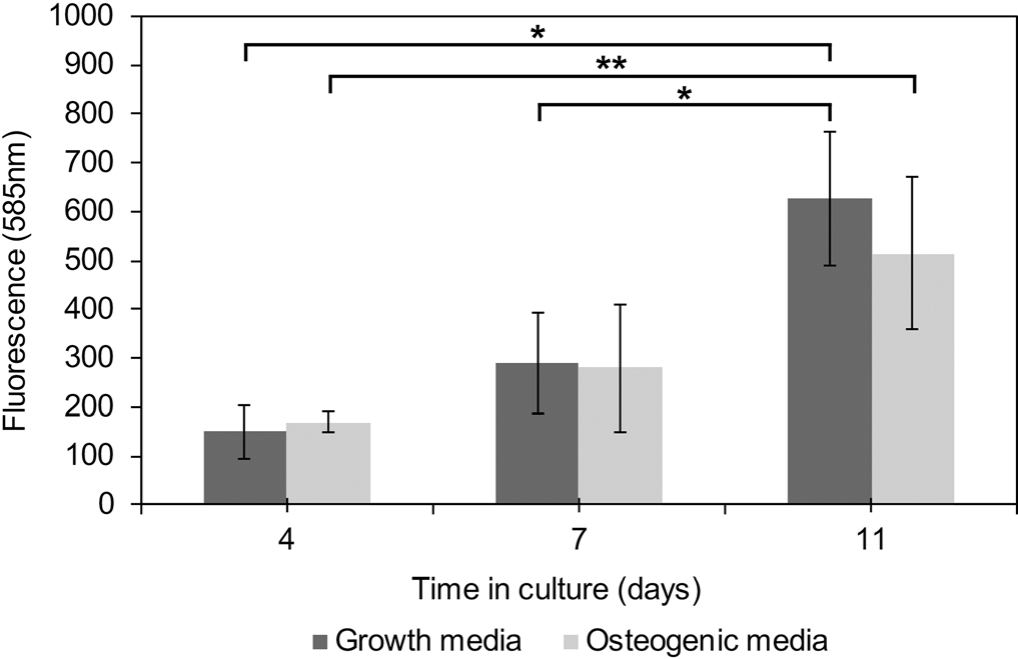
Cell metabolic activity for 11 days of cell culture on the microspheres via Resazurin assay, mean ± 95% confidence intervals. Similar growth profiles for both growth media and osteogenic media. ANOVA multiple comparison was used to determine no difference (p > 0.05) between each media at each time point. All graphs showing mean ± standard deviation (SD), n = 3.

**FIG. 5.**
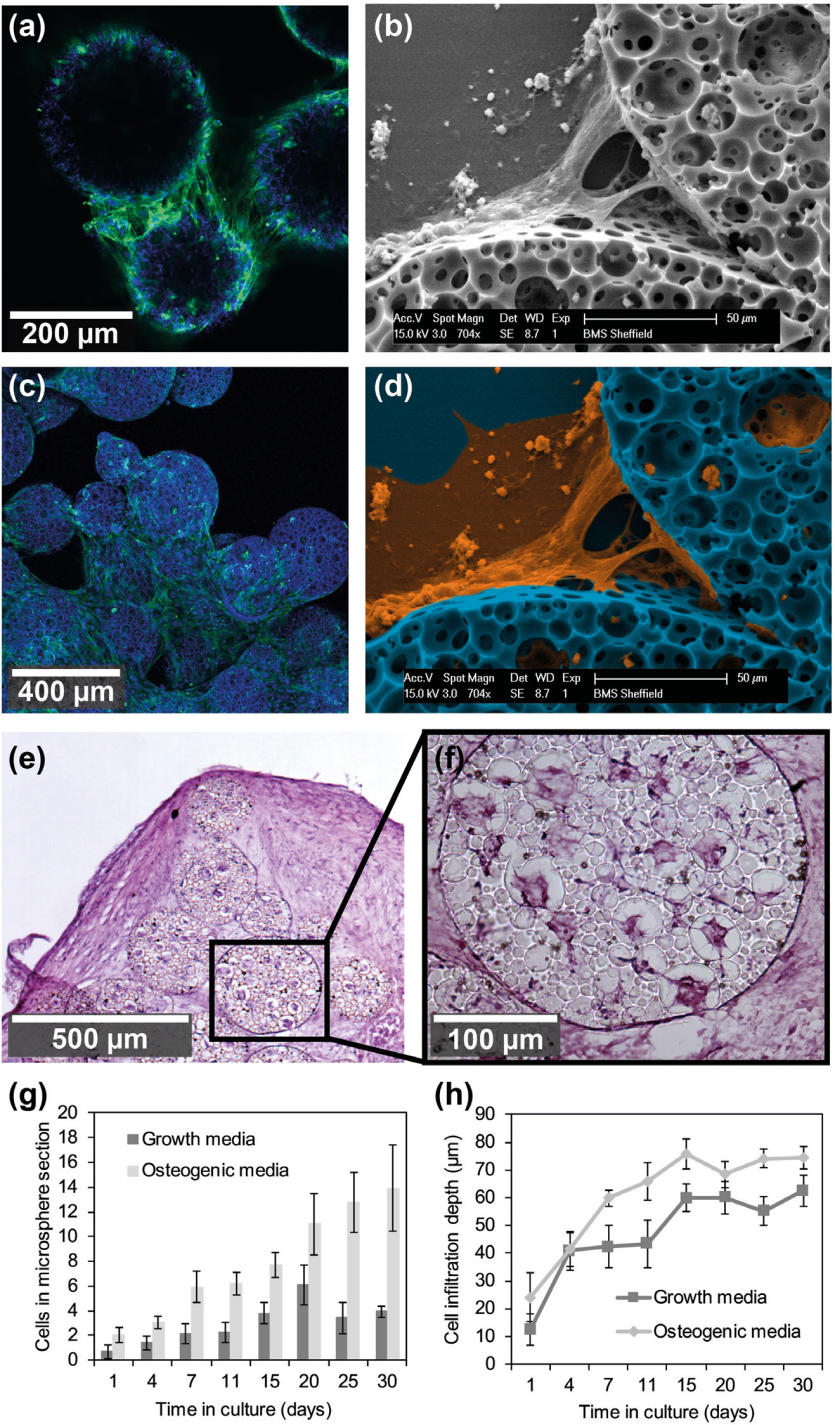
Initially many aggregations of a few microspheres were found at day 3 (a), and by day 14 (c), the cells had formed into a larger structure. Images [(a) and (c)] are confocal z-stack images of nuclei (blue) and actin (green). [(b) and (d)] SEM image of the 50 *μ*m thick section of polyHIPE microspheres fixed after culture in osteogenic media. (b) Non-processed, SEM image of the ECM binding two microspheres together along with the cells and ECM extending out beyond the microspheres. (d) False colour SEM image of (b) highlighting the ECM-resembling material in orange. (e) H&E stain of the 20 *μ*m thick section of microsphere-cell agglomeration, after 60 days in osteogenic culture medium. (f) Higher magnification image of a single microsphere from (e). (g) Plot of the number of cells found within a microsphere cross-section over the culture period, used as a measure of cell ingrowth into microspheres. (h) Plot of the cell depth of 5 cells closest to the centre of the microsphere, showing cell ingrowth into the microsphere as the time in culture increased. All graphs showing mean ± standard deviation (SD).

Cells were seeded on acrylic acid coated microspheres (40–600 *μ*m) prepared via CSTR, and their viability was measured at several time points using a resazurin salt assay (Fig. 4). A significant increase in cell activity was observed over the 11 days, particularly between days 4 and 11 for both media. No difference in cell activity was observed at any time point between the osteogenic and growth media used in culture. After the third day in culture, it was observed that the microspheres began to form aggregates with cells forming bridges between microspheres [Fig. 5(a)]. Viability increased up to day 11 by which point all the microspheres had been incorporated into larger aggregates of proto-tissue. A steady increase in cell viability was found on the acrylic acid coated polyHIPE material further establishing this material's potential in bone tissue engineering.

### Aggregate formation via cell matrix encapsulation of the microspheres

Images of microspheres taken at two time points using a confocal imaging system showed the formation of cell-initiated aggregations during *in-vitro* culture. It is possible to see both the increasing size of the aggregations and the increasing numbers of cells present on and around the structures. Initial formation of many smaller units of a few microspheres is observed at day 3 of culture. These smaller units gradually combine to form larger agglomerations over the 14 days in culture. The extracellular matrix (ECM) holding the microspheres together can be observed in Fig. 5(b) and in false colour in Fig. 5(d). The ECM spans the distance between the two microspheres with a fibrous appearance. Cells are observable within all the large pores of all the microspheres after 60 days in culture in osteogenic media [Figs. 5(e) and 5(f)]. To ensure a repeatable and controllable test of cell ingrowth monodisperse microspheres were used and cells were observed in increasing numbers inside the microspheres over the culture period [Fig. 5(g)]. The number of cells within microspheres cultured in osteogenic media increased at a faster rate than those cultured in growth media. There was comparatively less ingrowth observed into microspheres cultured in growth media over the entire experiment with internal cell numbers remaining consistent. Cells grew further into the microsphere over the course of the experiment as can be seen in [Fig. 5(h)] with cells being close to the centre point of 100 *μ*m after 30 days.

**FIG. 6.**
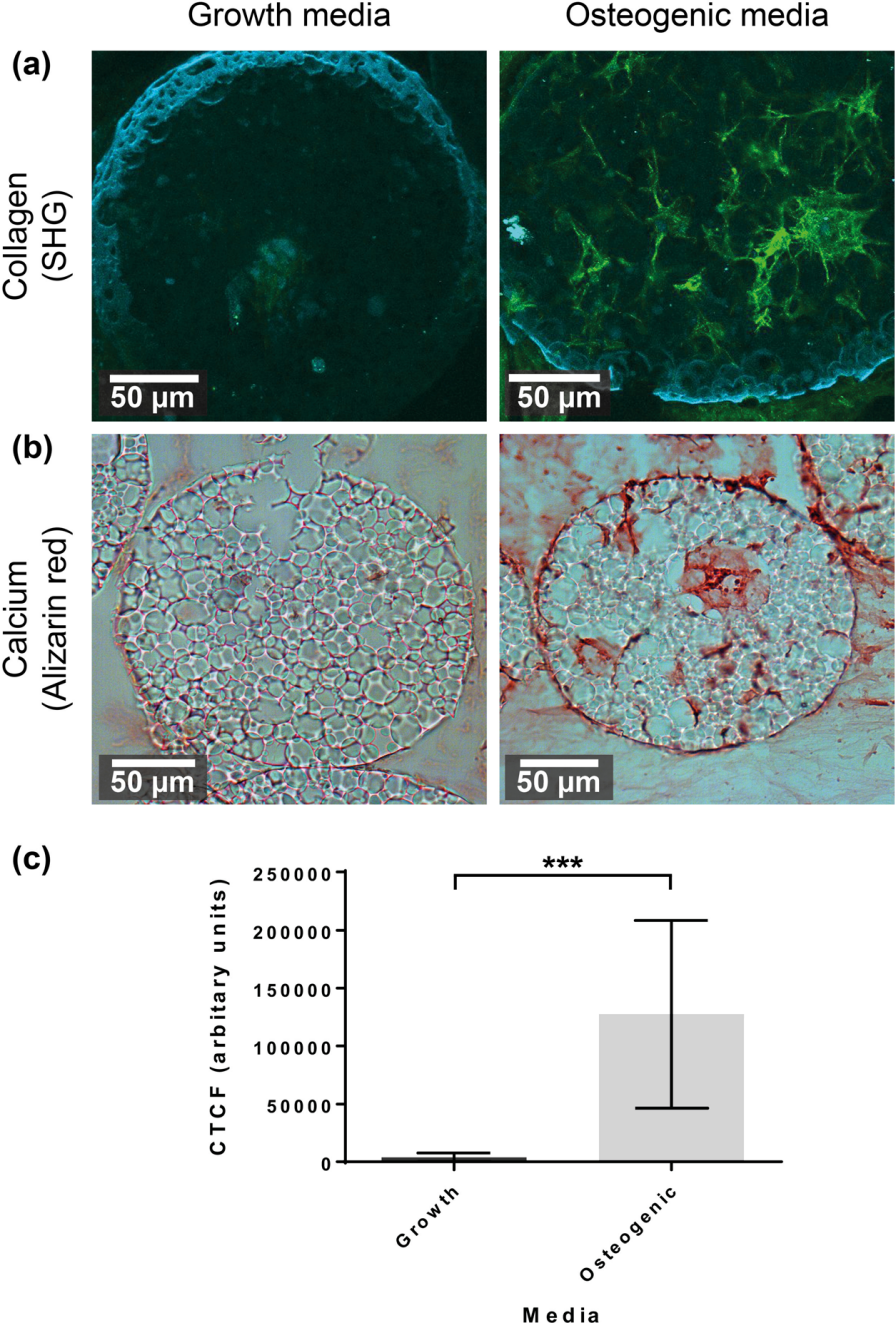
Sections of microspheres chemically or optically measured to assess the presence of calcium and collagen deposits. (a) Second harmonic generation using a confocal microscope is shown in green, and the superimposed image of the microsphere is shown in blue. (b) Alizarin red stain for calcium shows the presence of calcium within microspheres cultured in osteogenic media. (c) Graph of corrected total cell fluorescence (CTCF), a measure of the intensity of the SHG signal from both samples, showing a statistical difference. Analysed with T-test, showing mean ± standard deviation (SD).

Staining and optical analysis on sections of the microspheres showed the presence of calcium and collagen (Fig. 6). Second harmonic generation (SHG) confocal imaging showed the presence of mature collagen within the microspheres cultured in osteogenic media [Fig. 6(a)]. A measure of the total fluorescence showed a significantly higher amount in microspheres and cells cultured in osteogenic compared to growth media [Fig. 6(c)]. Alizarin red confirmed that an early bone-like matrix was formed, indicating deposits of calcium within the microspheres cultured in osteogenic media [Fig. 6(b)].

### Angiogenic potential of microspheres assessed in a chick CAM assay

A CAM assay was used to determine the ability of the microspheres to stimulate angiogenesis when implanted with and without cells. Microspheres with cells had been pre-cultured for 21 days before implantation and had formed into agglomerations similar to those found in Fig. 5. Microspheres without cells and hES-MP cells without scaffold support were also tested. After implantation and incubation for 7 days, the implantation site was exposed and imaged and the area was extracted for histological analysis.

**FIG. 7.**
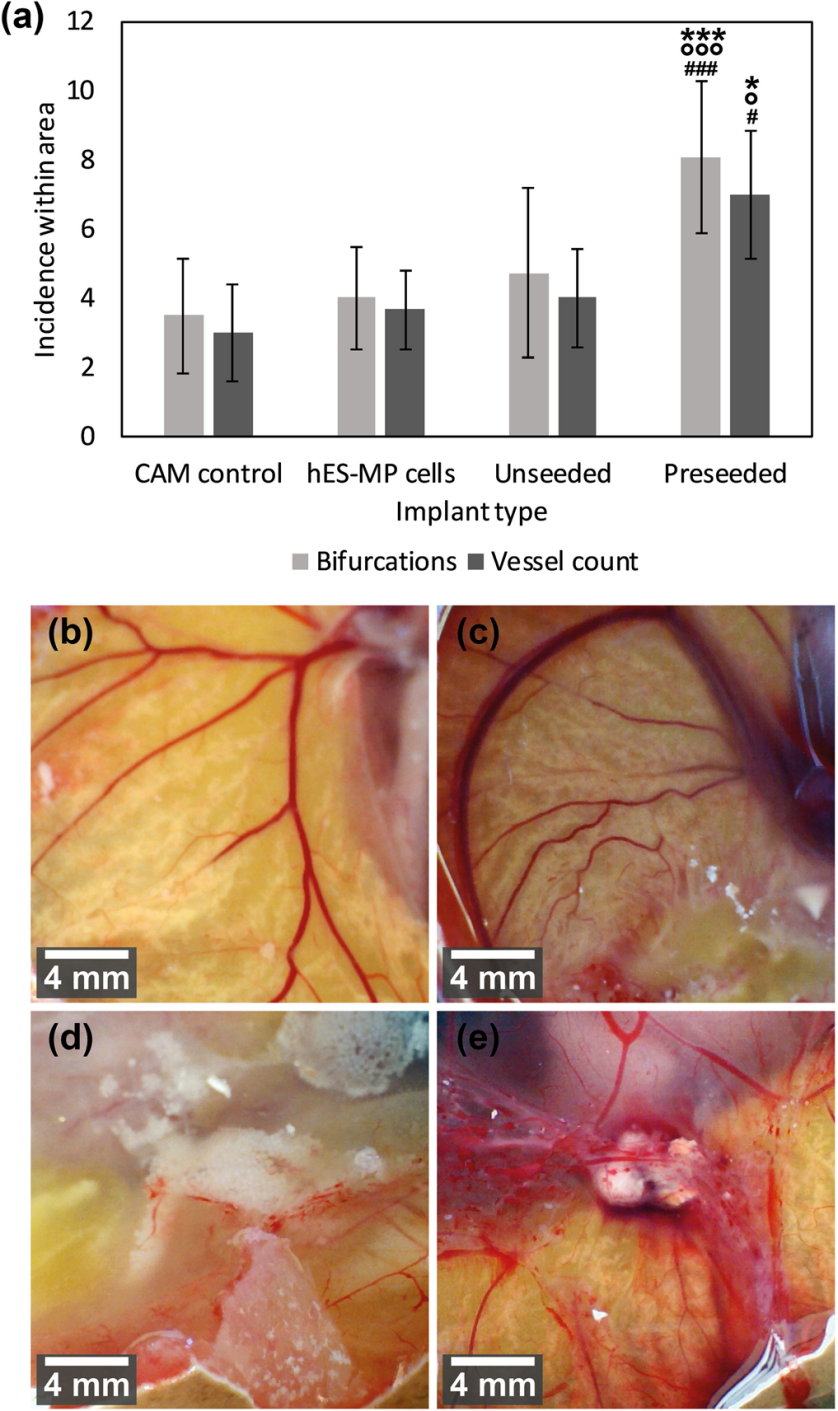
(a) Mean number of blood vessels and vessel bifurcations around the implant. Significant differences in the vessel number were found between the pre-seeded microspheres when compared to the other three implant types and control. Optical images from a handheld USB microscope of the implant site within the CAM models after 7 days of incubation. (b) Control was from a CAM which had been opened for implantation and then closed. (c) CAM seeded with hES-MP cells without microspheres. (d) Microspheres that were not pre-cultured with any cells. (d) Microspheres were seeded with hES-MP cells for 21 days before implantation into the CAM. One-way ANOVA, mean ±standard deviation. n = 6. Significance is indicated by the following symbols: * for significance compared to control, # for significance compared to hES-MP control, and ° for significance to unseeded microspheres.

The level of vasculature around microspheres pre-cultured with cells was significantly greater than that of either unseeded microspheres or the hES-MP cell culture (Fig. 7). The numbers of blood vessels and vessel bifurcations around the implant site were counted. The pre-seeded microspheres showed a significant increase in both the number of blood vessels present (which were approximately double those seen in the absence of cells) and the branches on each vessel when compared to all the other samples. This confirms the results observed visually in Figs. 7(b)–7(d). There was no statistical difference in the vessel number or bifurcations between un-seeded microspheres and control CAMs or hES-MP implanted CAMs.

### Cell migration into microspheres implanted in the CAM assay

**FIG. 8.**
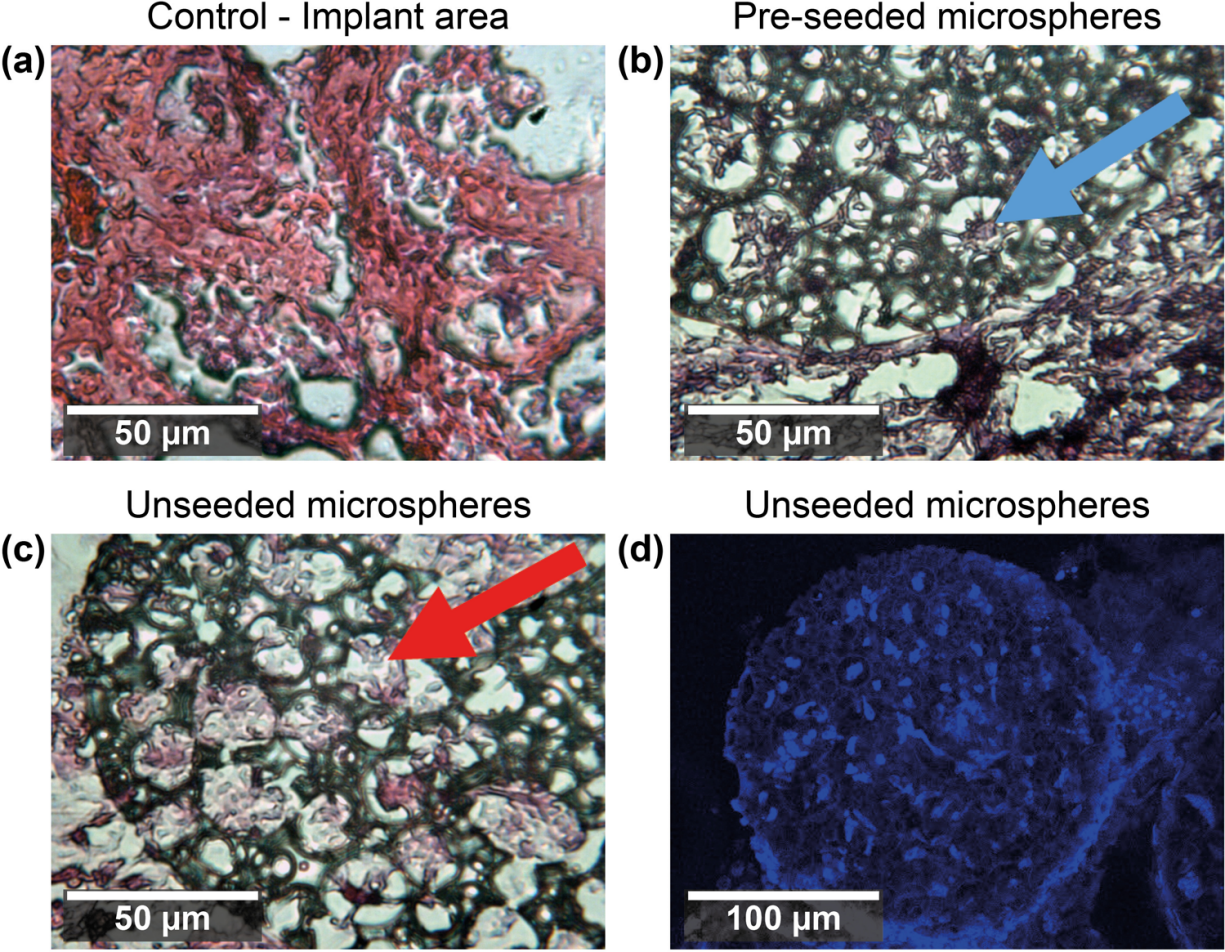
H&E staining of 10 *μ*m thick sections of scaffolds and tissue extracted from the implant area. (a) Staining of the implant area of the control CAM assay. (b) Images from the seeded microspheres, cells can be observed within the pores of (b). The blue arrow points to a cell within a pore, which from seeding and previous staining and morphology is expected to be an osteoblast. (c) Images of the unseeded microspheres embedded in CAM tissue. The red arrow points to a chicken cell which has entered an unseeded microsphere during the CAM culture. (d) DAPI staining of the nucleus material. Confocal z-stack images of a microsphere from the implant site which had been implanted into the CAM without the addition of any cells. Cells can be observed saturating the internal porosity.

Sections of microspheres extracted from the CAM assays after 7 days and stained with haematoxylin and eosin (H&E) or 4′,6-diamidino-2-phenylindole (DAPI) show cells within the pores of all microspheres, even those that were not implanted with hES-MP cells (Fig. 8). Microspheres implanted without cells showed ingrowth from cells in the CAM over the 7-day incubation period, and cells were present throughout the microsphere [Figs. 8(c) and 8(d)]. The cells seen are native CAM cells growing within the pores of the microsphere, as no other cells were added to these microspheres before implantation. A morphological difference can be observed between the cells within the pores of the microspheres, depending on the implantation condition (blue and red arrows, Fig. 8).

## DISCUSSION

This study shows that it is possible to manufacture highly porous microspheres to deliver MSC-like cells and stimulate angiogenesis, indicating that these microspheres can be developed for injection into complex non-healing bone fractures to accelerate healing. A major problem in the stimulation with the difficulty in healing bone fractures is the lack of a blood supply to support new bone growth. MSC cells can act as pericytes (helper cells) to stabilise microvasculature tube formation and sprouting.^31^ However, a methodology for delivering these cells and keeping them in a protective environment such that they can stimulate new blood vessel growth is needed. We report evidence based on the chick CAM assay that MSC-like cells delivered on their own did not deliver significant angiogenic benefits, but when delivered in highly porous microspheres, they stimulated new blood vessel growth.

The requirements for microspheres for bone repair are that they are highly porous, reproducible in their manufacture, capable of being injected, can support osteogenic cell growth, and stimulate angiogenesis. Therefore, we used a polyHIPE double emulsion microfluidic technique to manufacture the particles. While not degradable using the chemistry proposed here, this technology could be translated to degradable polymers, for example, recently highlighted biodegradable thiol-ene^32^ and polypropylene fumarate dimethacrylate-based polyHIPEs.^33^ Additionally, biodegradable porous particles have been produced by, e.g., emulsion templating (polypropylene glycol)^34^ and by freeze drying (gelatin).^35^

The polymer blend we used was previously reported by our group^36^ as having a stiffness of 28.4 ± 3.6 MPa. This choice of stiffness was selected to ensure maximum stiffness without the material becoming too brittle to handle and post-process. This material stiffness was aimed to support differentiation to osteoblasts.^29^ Our previous study demonstrated that the stiffer composition (25–50 MPa) of this material blend better supported higher osteogenic differentiation [as measured in alkaline phosphatase (ALP) production] compared to lower stiffness compositions (1–10 MPa). Furthermore, the study indicated that plasma coating with acrylic acid significantly increased ALP production. The microspheres received the same plasma coating treatment.

It was possible to control the porosity and pore size of the resulting polyHIPE material by altering conditions during the initial emulsion formation stage prior to the double emulsion step to form the microspheres. The stir rate of the solution during water addition and the temperature of this water were found to change the pore sizes observed in the polyHIPE material. The surface porosity of a polyHIPE has been shown to influence the cell attachment and cell differentiation of hES-MP cells.^37^ The internal pore size has also been shown as important in controlling cell differentiation.^38^ The internal porosity of these particles is importantly interconnected to enable ingrowth. This ability to change the pore size is a function of the emulsion stability, with more stable emulsions able to maintain smaller water droplets (increased surface area between the two phases). The emulsion stability is dependent on a range of factors including the shear rate applied to the solution during emulsion mixing and the temperature during the HIPE formulation.^39^ Larger voids and interconnecting windows in the polyHIPE can be created through a controlled destabilisation of the emulsion by increasing the temperature of the droplet phase to 80 °C.^40^

The T-junction microfluidic method produced a comparatively monodisperse size population of microspheres, whereas the CSTR produced a wider range of sizes. The CSTR production method relies on stirring the emulsion in an excess of water to produce the double emulsion. Production of microspheres in these turbulent flow conditions will result in a highly polydispersed sphere size. In contrast, the T-junction microfluidic method produces spheres via budding off in a highly controlled constant flow, thus greatly improving the sphere size distribution. Microspheres produced in a broad distribution of sizes would allow for a higher random packing density which could reduce the size of the voids between microspheres, and this could reduce the space available for vascularisation to occur. For microsphere populations featuring a narrow size distribution, the lower packing density would allow the engineering of pore diameters between the microspheres to best manage the void size between adjacent microspheres. While it is more difficult to produce a defined microsphere size distribution via the CSTR method, it is still possible to approximately control the median microsphere size. The microsphere sizes produced at a particular stir rate appear to form a skewed distribution as also noted by Zhang *et al.*^41^ Other groups similarly reported that the average microsphere diameter decreased with the increasing stirring rate.^42,43^ Producing microspheres via a microfluidic device however may not be suitable for less stable HIPE solutions. Indeed, Choi *et al.* demonstrated that a polylactic-co-glycolic acid (PLGA)-based emulsion began to separate out into multiple phases soon after formation and this phase separation was exploited to produce microspheres with variable pore sizes.^22^

Using either CSTR or T-microfluidics to form particles did not significantly alter the internal pore size of the resulting microsphere, as the pore size is determined at the HIPE formation stage. There was no significant difference between the internal porosity when the emulsion was subjected to different water stirring rates, or water temperature, during the w/o/w fabrication process. These observations concur with the results reported by Bou *et al.*^44^ This allows the control of the pore size independent of conditions or techniques used to tune the microsphere size.

In contrast, there is a clear difference in the surface pore architecture of the microspheres produced by the two methods. In the CSTR method, the emulsion droplets are continuously surrounded by water, while in the T-junction microfluidic set-up, the emulsion is dispensed through a small diameter (0.15–0.5 mm) metal syringe, which will influence the surface porosity of the HIPE. This surface roughness plays an essential role in the attachment of cells.^45^ Both the small diameter of the syringe and the contact of the HIPE with the metal can influence the final surface porosity of the produced polyHIPE microspheres. Indeed, the surface porosity of polyHIPEs is highly affected when curing them on different surface energy materials.^24^ Surface destabilisation of the emulsion could be occurring at the emulsion syringe interface depending on the surface energy, where a preferential wettability of the oil phase may be creating a thin monomer film around the HIPE droplet, resulting in a surface skin upon polymerisation. The surface porosity of the polyHIPE is dependent on the material that the HIPE is in contact with during polymerisation.^24^

This investigation found that tailored porous microspheres were able to support hES-MP cell growth over 11 days in culture. Microspheres were found to aggregate over time as cells and extracellular materials were observed binding the microspheres together. Multiple small aggregate nuclei were formed after a few days, which then increased in size, as single microspheres and other small aggregates combined. Aggregates remained as a solid structure and cells continued to proliferate on the exterior. The process of cells and microspheres combining after a period of time to form large cell-microsphere aggregates has been observed by a number of studies.^46,47^ Reis *et al.* have shown that it is possible to form aggregates in a faster fashion by increasing the cell and microsphere contact area by growing them within an Eppendorf tube.^15^ This suggests that forcing microspheres together, such as would occur within a closed wound, would allow aggregate formation to occur more rapidly than observed *in vitro*.

Cells were observed within the microspheres' internal porosity after culturing with hES-MP cells in osteogenic culture media. These cells have migrated into the microsphere from the external surface, passing through the interconnecting regions between the pores. Cells were observed growing in the centre of microspheres, up to 90 *μ*m from the surface. Sections of the samples after 60 days show that almost every pore larger than 15 *μ*m has been inhabited by a cell. We have calculated that after 30 days in osteogenic media, the total number of cells per microsphere is roughly 100. This number was obtained by using the number of cells in a 20 *μ*m thick slice and extrapolating the cell number to the total volume of the microsphere. Studies have demonstrated that the optimal pore size for ingrowth is >100 *μ*m (Refs. 48 and 49), whereas the average porosity within the polyHIPE studied here was 25 *μ*m, with few interconnecting channels between the pores being larger than 7 *μ*m. Lu *et al.* studied the culture of osteoblasts in porous ceramics and found that within their interconnected porous system, osteoblasts/bone ingrowth required interconnecting pores larger than >20 *μ*m in diameter^50^ which is larger than those in the polyHIPE material. Previous studies on hES-MP cells grown on polyHIPEs have focussed on scaffolds with larger pore sizes.^51^ Smaller pores (∼50 *μ*m) are reported to stimulate the production of osteoblast-like cells *in-vitro*.^30^ Consequently, we concentrated on microparticles with smaller pore sizes, comparable to the polypropylene glycol-based particles studied by Moglia *et al.*,^34^ to facilitate osteogenic differentiation *in-vitro*. Intranuovo *et al.*^52^ demonstrated in their paper that a coating gradient exists when using plasma based coating techniques in large (1 cm diameter by 5 mm height) porous scaffolds (∼250 *μ*m pore sizes). This can be exploited to enhance the ingrowth of cells within these large scaffolds. In our case, the individual scaffolds are much smaller (200 *μ*m spheroids), and it is unlikely that a discernible gradient is established in these spheroids.

hES-MP cells cultured on microspheres within osteogenic media were found to produce both mature collagen and calcium after 28 days in culture, which indicates that the cells have become osteoblastic in nature.^53,54^ Using SHG confocal imaging, collagen was shown within the pores of the microsphere. Alizarin red staining of the microspheres also showed deposits of calcium within the pores of the microspheres. These pores are known to contain cells, as can be seen in the H&E staining, and this suggests that the cells that have entered the microspheres have begun to differentiate into osteoblasts. When cultured in growth media, cells were not observed within the microsphere pores and no osteoblast indicators were observed.

An enhanced vascularisation response was observed in the CAM assay after 7 days for microspheres pre-cultured with hES-MP cells in osteogenic media, when compared to either cells or microspheres in isolation. This suggests that cells bound to the microspheres act synergistically to stimulate angiogenesis. However, microspheres contained cells cultured in osteogenic media, whereas the cells in the control were cultured in growth media, and therefore, the cells introduced to the CAM assay are likely to be different. The microspheres were agglomerated within a microtissue, and it is likely that a significant sub-population of cells reside within a hypoxic environment. This could stimulate the endogenous release of angiogenic growth factors from the hypoxic cells.^55^ It is also possible that the microspheres provide physical protection to cells, which would keep cells in a concentrated area instead of dispersing in the CAM. Early differentiation (days 4–14) of MSC cells into osteoblasts is typically marked by an upregulation of vascular endothelial growth factor (VEGF) from the cells.^56^ This upregulation may be partially responsible for the increased vascular response, in addition to the potential presence of hypoxic cells. We can conclude that while it is not the microspheres themselves which cause the angiogenic response, they are potentially involved in enabling the cells to promote blood vessel formation.

The packing of spherical objects such as microspheres will include voids although these gaps are unlikely to be large enough for blood vessel ingrowth. In this regard, as the microspheres are not fixed in place, it could be possible for the growing vasculature to displace the microspheres to grow. The microspheres were designed to be 200 *μ*m in diameter to ensure that cells would be able to receive nutrients as they were well within the diffusion range.

In the CAM assay, microspheres implanted without cells did not produce a vascularisation response. However, the microspheres were found to recruit local cells from the developing chick, which fully infiltrated the internal porosity of the scaffold over the 7-day study. This must have occurred through the small, >7 *μ*m diameter, interconnecting windows between the pores. This *in-vivo* finding suggests that empty microspheres may recruit cells from within the human body if implantation occurred without pre-culturing with cells.

## CONCLUSION

Two methods of manufacturing microspheres were compared which differed in the particle size distributions but both achieved microspheres with an extensive interconnected pore distribution. The particles were coated with acrylic acid to improve hES‐MP adhesion and consequent proliferation. hES‐MPs were observed migrating into the microspheres and showed indications of differentiation into osteoblasts. These particles preloaded with hES‐MPs were found to be angiogenic in a chick CAM assay. Interestingly, this result was only observed when hES‐MPs and microparticles were used in combination. We suggest that this is a promising approach for an injectable scaffold to deliver MSC and inducing angiogenesis in non-healing bone fractures or in reconstructive surgery.

## METHODS

All chemicals were purchased from Sigma-Aldrich, UK, and used as supplied unless otherwise stated.

### High internal phase emulsion (HIPE) preparation

A HIPE is formed by mixing two immiscible liquids to form a stable emulsion, and the EHA/IBOA copolymer HIPE was prepared according to a published procedure.^29^ Briefly, a HIPE was prepared by mixing the monomers isobornyl acrylate (IBOA, 3.66 g) and 2-ethylhexyl acrylate (EHA, 1.56 g) with the crosslinker trimethylolpropane triacrylate (TMPTA, 1.41 g) using an overhead stirrer. The surfactant Hypermer B246 (0.21 g, Croda) was added, and the solution was mixed until it was dissolved. The photoinitiator diphenyl(2,4,6-trimethylbenzoyl)phosphine oxide/2-hydroxy-2-methylpropiophenone blend (0.35 ml) was added before the addition of water.

To prepare a HIPE with an aqueous content of 80%, 28 ml of deionised water was added over 5 min in a drop-wise fashion into a 100 ml capacity beaker containing the oil phase, stirred at a specific stirring speed using an overhead stirrer (Lab Egg, IKA). The emulsion was stored in an amber glass vial (Supelco) and used within 6 h of preparation. The stirring speeds used to investigate processing conditions were 320, 540, 765, 870, and 1260 rpm. To investigate the effect of temperature on porosity, the aqueous temperature was stabilised at 4, 15, and 30 °C before addition to the monomer.

### PolyHIPE microsphere manufacture via CSTR

Using a stirred tank reactor is a popular method of producing particles of one phase in the second immiscible phase. A 100 ml capacity beaker (50 mm diameter and 70 mm height) containing deionised water (40 ml) was set stirring at a defined rate using an overhead stirrer (Lab Egg, IKA). HIPE (2 ml) was added to the beaker in a drop-wise fashion with continuous stirring at room temperature. The resulting double emulsion (w/o/w) was left stirring for 2 min (350 rpm). Stirring was subsequently stopped, and the microspheres were cured immediately using the UV output of a mercury lamp (Omnicure S1000, 100 W).

### PolyHIPE microsphere manufacture via T-junction microfluidic

As the alternative fabrication method, a microfluidic system was developed based on the one previously described.^25^ Briefly, a small internal diameter (0.15–0.51 mm, Nordson EFD) dispensing tip was used to inject the photocurable HIPE into a 6 mm diameter silicone tube (Advanced Fluid Solutions) through which a continuous flow of deionised water was driven via a peristaltic pump (Masterflex L/S tubing pump, Cole-Palmer). The resulting droplets were immediately cured by the UV output of a mercury lamp (Omnicure S1000). The monomer flow rate was 3 ml/h (syringe pump, GeniePlus, Kent Scientific), while the water flow rate was set at 300 ml/min. A continuous stream of microspheres was produced, which were then collected in a 40 *μ*m sieve (40 *μ*m cell strainer, BD Falcon). The system ran for 30 min to stabilise before any microspheres collected were utilised. The flow rate was altered to the following settings to examine the water flow rate impact on the microsphere size and distribution: 125, 250, 375, 500, 620, and 745 ml/min.

### Imaging of polyHIPE samples

SEM analysis was performed on sectioned and whole microspheres along with sectioned microspheres cultured with human embryonic stem cell-derived mesenchymal progenitor (hES-MP) cells. Samples were mounted on aluminium stubs using adhesive carbon tabs and sputter coated with gold (SC500, emscope) with a current of 15 mA for 2 min at 0.05 atm. Images were then acquired using a scanning electron microscope (Philips/FEI XL30 ESEM) operating with an electron beam energy of 15.0 kV. To determine the pore size, microspheres were sectioned using a cryostat (Leica CM1860 UV) to 40 *μ*m sections in Tissue Freezing Medium (Leica) which was allowed to evaporate for mounting and gold coating. At least 50 pore sizes were measured from each of the 3 SEM micrographs of a sample using ImageJ software (NIH). At least two independent samples were analysed.

PolyHIPE microspheres were imaged using a reflected light optical microscope (Miotic B5 professional series) and measured in ImageJ using a calibrated scale from a stage micrometre (imaging apparatus). All microspheres in each image were measured using ImageJ until n > 200. A histogram was used to compare microspheres manufactured by both methods.

### Plasma polymerisation of acrylic acid onto the polyHIPE material

Data from our laboratory on scaffolds of similar chemistry indicated that plasma coating with acrylic acid enhances cell adhesion on the HIPE material.^29,57^ Therefore, in preparation for cell culture, the surface chemistry of the polyHIPE microspheres was modified by inductively coupled plasma polymerisation of acrylic acid. The plasma polymerisation was carried out in a custom-made apparatus consisting of a cylindrical borosilicate glass chamber with stainless steel endplates connected to the vacuum pump (RV8, Edwards). Chamber pressure was monitored by a Pirani gauge (APG-L-NW25 Edwards) and manually controlled by a needle valve regulating the flow of monomer vapour (Edwards LV10K). The flow of monomer vapour (acrylic acid) was established through the chamber at 2.4 cm^3^/min. The electromagnetic field was generated by a coil wrapped around the chamber connected to a radiofrequency generator (Coaxial power systems limited). The power to the coil was manually adjusted to 15 W, and the polymerisation was allowed to proceed under these conditions for 20 min. Microspheres were spread out evenly on aluminium foil within the chamber.

### Preparation and culture of hES-MP cells

hES-MP cells (Y10090 Cellartis hES-MP 002.5) were maintained and passaged in a growth medium composed of alpha minimum essential medium eagle (MEM) basal medium (αMEM, Lonza) supplemented with human basic fibroblast growth factor (bFGF) at 4 nM (Life technologies), 100 mg/ml penicillin-streptomycin, 10% foetal calf serum (FBS, Labtech), and l-glutamine at 2 mM concentration. hES-MP cells were cultured in T-75 flasks that had previously been coated with 0.1% bovine gelatine solution for 20 minutes before rinsing. Separate media were prepared with supplements to promote osteogenesis. Ascorbate 2 phosphate at 50 *μ*g/ml, beta-glycerol phosphate at 5 nM, and dexamethasone at 10 nM were added to induce osteogenic differentiation termed “osteogenic media.”

### hES-MP cell *in-vitro* culture on polyHIPE microspheres

Microspheres were tested to investigate their ability to support a cell population and to determine differentiation. The samples were sterilised in 70% ethanol for 30 min, then washed 3 times in deionised water, and soaked in αMEM media for 1 h. 100 000 hES-MP cells were suspended via gentle pipetting and added to a T-25 flask (Greiner bio-one) containing 0.1 g of microspheres in designated media (growth and osteogenic media). The T-25 flask was kept vertical to allow cells the longest opportunity to attach to the microspheres. The flask was placed on a rocking platform for 45 min at 12 oscillations per minute. The T-25 flasks were then incubated for a further 2 h at 37 °C before microspheres were transferred to a new T-25 flask. The microspheres were then rinsed with phosphate buffered saline (PBS) to remove unattached cells before adding new medium. Media were replaced every 2 days by removing 80% of the medium and replacing it with fresh medium. Cells were cultured for 11 days, and samples were taken at days 4, 7, and 11 for confocal microscopy and cell activity measurements using Resazurin salt assay.

### CAM preparation, implantation, and extraction

The CAM assay was used to assess the vascularisation response of the microspheres in an *in-vivo* environment. This assay was performed according to the published procedure^58^ and consistent with the Home Office, UK guidelines. Fertilised chicken eggs (*Gallus domesticus*) purchased from Medeggs were incubated from day 2 of fertilisation until day 8 at 37 °C in a humidified egg incubator (R-COM Suro20). At day 8, a window was cut into the shell of the egg (5 mm^2^) and the implants were injected into the opening using a 5 ml syringe with a 1.1 mm internal diameter needle tip. Masking tape was used to secure sterilised (in ethanol, 30 min) parafilm over the implantation site to prevent infections.

Each egg was implanted with one of the following samples or controls. 0.5 g of polyHIPE microspheres cultured as above for 3 days in osteogenic media with hES-MP cells were washed and injected in PBS for implantation. 0.5 g of polyHIPE microspheres soaked in osteogenic media for 3 days without cells were washed and injected in PBS for implantation. 100 000 hES-MP cells cultured in growth media (P4) were injected in PBS into the egg. A control was used where the egg was opened and then resealed without the addition of any foreign objects.

The chicken eggs were incubated until day 14 when the scaffolds were retrieved and the eggs were terminated. The chicken eggs were removed from the incubator immediately prior to processing. Angiogenesis was quantified by light microscopy just before scaffold retrieval (Miotic) and using histological images of the retrieved scaffolds. Data on the density and bifurcations of the blood vessels were obtained by analysing a 2 cm^2^ area around the implant over a series of images.

### Resazurin salt assay for assessing hES-MP cell activity on microspheres

Cell activity of hES-MP cells on polyHIPE microspheres was determined using the Resazurin salt assay. Resazurin sodium salt (5 mg/ml in PBS, 4 ml) was added to each T-25 flask for 4 h at 37 °C with the flask turned onto one side to avoid measurements from cells deposited on the base plastic. The solution was removed with 200 *μ*l added to a 96 well plate in triplicate. Fluorescence was measured using a fluorescence plate reader (FLX800, BIO-TEK Instruments, Inc.) with excitation at 570 nm and peak emission measured at 585 nm.

### Histology of *in-vitro* and CAM samples

Histology was performed on microspheres cultured *in-vitro* with hES-MP cells and with scaffolds retrieved from the CAM assay. Scaffolds were fixed in 3.7% paraformaldehyde in deionised water for at least 30 min. The samples were placed into cryosectioning moulds and surrounded by optimal cutting temperature (OCT) compound (Leica). These were frozen by submersion into a bath of liquid nitrogen. Sections were cut with the cryostat Leica CM1860UV (Leica). Samples were then stained with haematoxylin and eosin solutions (H&E), using the standard protocol for frozen sections. Slides were then imaged using a light microscope (Motic).

### Fluorescent labelling of f-actin and nuclei of cells on scaffolds for imaging

Samples from both the *in-vitro* culture of hES-MP cells on microspheres and the scaffolds from the CAM assay were stained for f-actin and cell nuclei for fluorescence imaging. Samples were washed 3 times in PBS, fixed in 3.7% formaldehyde solution for 50 min, and then washed with PBS. Samples were permeabilised for 15 min in a solution of PBS and 0.1% triton X-100 and then washed 3 times with PBS. DAPI and fluorescein isothiocyanate (FITC)-conjugated phalloidin stains were made up at a concentration of 1:1000 in PBS and added to the samples for 1 h at 4 °C. Samples were washed with PBS, then stored in PBS, and imaged using a confocal microscope.

### Confocal microscopy of polyHIPE microspheres from culture

Microspheres cultured both *in-vitro* with hES-MP cells and those removed from the CAM assay were imaged using a confocal microscope. Images of 1756 × 1756 and 1024 × 1024 pixels were obtained using a Zeiss LSM 510META upright confocal microscope and either a 10× objective (Achroplan 10×/0.3 W, Carl Zeiss ltd) or a 40× objective (Achroplan 40×/0.75 W, Carl Zeiss Ltd). DAPI was excited using a 760 nm two-photon Ti-Sapphire laser (16% transmission) and emission detected between 435 and 485 nm. FITC Phalloidin was excited using a 488 nm laser (4% transmission) and emission detected above 505 nm. Z-stack images (1024 × 1024 pixels) were obtained using the same settings as single plane images, but repeated images were obtained of the same area, translated 11 *μ*m in the z direction after each capture.

### SHG collagen imaging

Second harmonic generation (SHG) is a fluorescent imaging technique allowing the imaging of mature collagen fibres within a sample. Samples were sectioned using microtome (40 *μ*m thick) from frozen sections of day 30 microsphere cultures. Samples were from microspheres cultured in growth media and osteogenic media. An oil immersion lens (×40) was used, and the sample was excited with a Ti-Sapphire 2-photon laser at 950 nm and the second harmonic generated signal detected between 469 and 480 nm. Images were taken at 512 × 512 pixels.

The SHG images of collagen were analysed to determine the significant difference in intensity between samples. The images for the analysis had been maintained at original intensity for comparison. ImageJ was used to measure fluorescence. ImageJ was used to find the area, integrated density, and the mean grey value of the regions of interest (microsphere location). Control regions were obtained from a non-fluorescent area. The corrected total fluorescence (CTF) was calculated by the following formula:

CTF=Integrated Density−(Area of selected fluorescence×Mean fluorescence of background readings).

### Alizarin red stain for calcium

The presence of calcium deposits is detected using an alizarin red stain. 1 ml of 1 mg/ml alizarin red in dH_2_O, adjusted to pH 4.1 by adding ammonium hydroxide, was added to the sample for 20 min. The sample was washed with dH_2_O until all unstained dye was removed. Samples were then rehydrated as above and then fixed.

### Measuring cell ingrowth as a function of time

To understand the behaviour of cell ingrowth into the microspheres over time in culture, a time course study was conducted. Uniform microspheres were produced to keep the diameter of microspheres constant. Samples were taken at days 1, 4, 7, 11, 15, 20, 25, and 30 fixed in 3.7% formaldehyde solution for 1 h and then sectioned and stained with H&E as above. The number of cells in each microsphere section was counted; cells at the surface were not included in the count. The 5 cells closest to the centre point of 100 *μ*m were measured using imageJ from the middle of the cell to the closest edge. Samples were left in osteogenic media until day 60 for sectioning and imaging.

### SEM preparation of hES-MP cell culture on polyHIPE microspheres using hexamethyldisilazane

Cells within sectioned microspheres underwent SEM analysis to determine the cell structure and location within the microsphere. After samples were sectioned and placed on 12 mm glass coverslips, PBS was used to remove the tissue freezing media. Samples were then treated with the following solutions to dehydrate the sample for SEM: 15 min in each solution of 35%, 60%, 80%, 90%, and 100% ethanol in distilled water, hexamethyldisilazane (HDMS) and ethanol (1:1 weight concentration) for 1 h, and then 100% HMDS for 5 min twice. Hexamethyldisilazane was then removed, and the samples were allowed to air-dry for 1 h. Samples were then gold coated and imaged using an SEM.

### Statistics

Statistical analysis was performed with GraphPad Prism using two-way analysis of variance (ANOVA) and plotted as mean ± standard deviation, unless otherwise stated. For *in-vitro* polyHIPE cultures with hES-MP cells, n = 3. For *In-vivo* CAM assay, n = 6. Data were tested for normal distribution using GraphPad Prism. A statistical correction factor of 2/√3 was used to derive the actual values of the pores from the measured diameters.^59^ This corrects for the measured diameter of a pore not being the actual midpoint of the pore, as a section will not cut each pore directly into half.

### Ethics approval

Care was consistent with the Home Office, UK guidelines. Ethics approval is not required.

### Data availability

The datasets generated during and/or analysed during the current study are available from the corresponding author on reasonable request.
